# Target-directed motor imagery of the lower limb enhances event-related desynchronization

**DOI:** 10.1371/journal.pone.0184245

**Published:** 2017-09-19

**Authors:** Kosuke Kitahara, Yoshikatsu Hayashi, Shiro Yano, Toshiyuki Kondo

**Affiliations:** 1 Department of Computer and Information Sciences, Tokyo University of Agriculture and Technology, Koganei, Tokyo, Japan; 2 Biomedical Engineering, School of Biological Sciences, University of Reading, Reading, United Kingdom; University of Minnesota, UNITED STATES

## Abstract

Event-related desynchronization/synchronization (ERD/S) is an electroencephalogram (EEG) feature widely used as control signals for Brain-Computer Interfaces (BCIs). Nevertheless, the underlying neural mechanisms and functions of ERD/S are largely unknown, thus investigating them is crucial to improve the reliability of ERD/S-based BCIs. This study aimed to identify Motor Imagery (MI) conditions that enhance ERD/S. We investigated following three questions: 1) whether target-directed MI affects ERD/S, 2) whether MI with sound imagery affects ERD/S, and 3) whether ERD/S has a body part dependency of MI. Nine participants took part in the experiments of four MI conditions; they were asked to imagine right foot dorsiflexion (F), right foot dorsiflexion and the sound of a bass drum when the sole touched the floor (FS), right leg extension (L), and right leg extension directed toward a soccer ball (LT). Statistical comparison revealed that there were significant differences between conditions L and LT in beta-band ERD and conditions F and L in beta-band ERS. These results suggest that mental rehearsal of target-directed lower limb movement without real sensory stimuli can enhance beta-band ERD; furthermore, MI of foot dorsiflexion induces significantly larger beta-band ERS than that of leg extension. These findings could be exploited for the training of BCIs such as powered prosthetics for disabled person and neurorehabilitation system for stroke patients.

## Introduction

In rapidly aging societies, the number of stroke patients living with physical impairments is increasing. To support and improve their quality of life (QOL), various motor assistance devices have been developed to restore the affected motor function. As one of them, Brain-Computer Interfaces (BCIs) have attracted much attention since it provides a communication channel from a human to a computer that translates brain activity into sequences of control commands for the assistance devices.

In most BCI research, an electroencephalogram (EEG) is accepted as a promising noninvasive communication channel. Indeed, EEG-based BCIs have been demonstrated to control information and communication technology (ICT) systems and robots (e.g., exoskeletons [1], powered wheel chairs [2], and computer-aided spellers [3]). Mental rehearsal of physical movement tasks or motor imagery (MI) has been widely used to train EEG waves for accurate control of BCIs linked to neuroprosthetics and other motor assistance devices. Control of MI-based BCIs can be acquired by neurofeedback training. For stroke patients with the paralyzed limbs, BCI is capable of bypassing the normal motor output pathways and directly translating brain signals into control commands. Brain control of such devices could be further improved by incorporating feedback signals to the somatosensory cortex within the MI, thereby creating a functional sensory-motor closed loop. In the field of neurorehabilitation, BCIs could restore the motor function of stroke patients with hemiparesis. Further, recent work has shown that motor rehabilitation during the acute stages can mitigate lasting motor impairments [4–8].

Event-related desynchronization/synchronization (ERD/S) [9] is an electroencephalogram (EEG) feature, and has been widely used for the BCIs purpose. The ERD is defined as a decrease of EEG power relative to a preceding rest state within mu (8–13 Hz) and beta (14–30 Hz) frequency bands correlated with Motor Execution (ME), Motor Imagery (MI), and Motor Observation (MO), while ERS is a rebound of beta-band power relative to rest state after termination of ME/MI/MO. He, B. et al. introduced the recent BCI technologies in detail from the steady-state visual evoked potential (SSVEP)-based BCI and P300-based training that require minimal training, to the Sensory-Motor Rhythm-based BCIs that require much longer training periods [10]. As more active intervention with the patients, the emerging neuromodulation technologies which modulate a certain part of the brain during the motor tasks should be paid attention. Application of anodal transcranial direct current stimulation (tDCS) during motor tasks could result in improved learning and performance [11]. This is a promising result, as a means to deliver the modulation by tDCS to strengthen the favorable brain activities during the motor tasks, also leaving us a chance to study associated memory and other cognitive aspects in the future. Realizing the importance of the mental states or the mindfulness, recent Mind-body awareness training (MBAT) in the form of yoga and meditation could enhance the subject’s ability to control the SMR signals, improving the overall performance [12]. Regarding the beta-band ERD, Yuan et al. summarized the recent BCI studies built on the rhythmic activity over the sensory-motor cortex, providing the comprehensive understanding over the different frequency range (alpha, beta, and gamma frequency) and their usage in a wide range of the BCI paradigm [13].

In addition to presenting features useful for signaling, ERD/S is somatotopic; for instance, ME/MI of right hand movement is reflected by ERD/S in the left sensorimotor area (position C3 of the international 10–20 system). Thus, we can discriminate the body region of MI (e.g., right or left hand) using a classifier [5, 6, 14] such as a support vector machine (SVM) [15]. In neurorehabilitation, it is crucial to detect the MI online with high accuracy, however, controlling ERD to reliably reflect the appropriate mental images (for desired movement) is a difficult skill to master, and is strongly dependent on individual MI ability [8, 16, 17]. It is known that the use of ERD/S induced by MI requires efficient and effective neurofeedback training. However, training programs for BCI control are further hampered by a lack of insight into the functional and physiological mechanisms of MI induction. Thus, this study aimed to identify effective MI training conditions that can enhance the generation of ERD/S. Effective training is expected to increase ERD/S intensity, thereby improving the accuracy of BCI classifiers and reducing the training period required to effectively use the BCI. It has been known for several decades that ERD can also be triggered by the observation of an others’ action [18]. The recent discovery of mirror neurons, a set of motor neurons that discharge during both action execution and observation, has shed a new light on the mechanisms underlying cortical motor rhythm changes during execution and observation [19]. Avanzini et al. reported that upper limb movement execution activated a larger cortical area when performed while viewing goal-oriented upper limb movement compared to non-target upper limb movement [20]. Further, this study also revealed greater ERD for target-directed movements relative to non-target-directed movement.

Numerous studies on sensorimotor rhythms (SMRs) have examined functional and physiological mechanisms of upper limb MI. However, it is also necessary to investigate lower limb MI for BCI control. Li et al. [21] asked subjects to imagine leg flexion/extension alone or while watching a video clip of goal-directed lower leg movement (kicking a ball), and found that watching the goal-oriented motion enhanced ERD during MI. However, these results do not clarify the effect of goal-directed MI on the ERD generation without any feedback stimulation, such as a video clip of the target task. The effect of an imagined goal could be critical for practical BCI use in daily life (i.e., outside of a laboratory or hospital). To examine target-oriented MI training using only an imaged target, ERD strength must be measured solely in an independent evaluation session [22].

Motor imagery training should be enhanced by an effective set of stimuli that close the loop between the motor intention and the sensory feedback. We speculated that observation of goal-oriented motion (motion towards a target) would benefit MI production. This notion is further supported by our previous study showing that dynamic visual stimuli of the forearm presented during four continuous BCI training days significantly improved generation of MI-associated ERD compared to static visual stimuli of the forearm [23].

A previous study also explored the feasibility of an auditory BCI in which subjects learned to control SMR amplitude [24]; however, ERD/S enhancement by auditory imagery has not been examined during MI training. Therefore, we investigated whether mental rehearsal of a sound associated with the result of the intended motion can enhance ERD/S. Furthermore, although previous studies have focused on discrimination of different MI tasks [25, 26], no one has investigated ERD/S during MI of different lower limb joints (i.e., ankle and knee). Therefore, we conducted systematic MI training experiments to investigate the following three questions: 1) whether target-directed MI affects ERD/S, 2) whether MI with sound imagery affects ERD/S, and 3) whether ERD/S differs with the body part of MI.

## Materials and methods

### Subjects

Nine right-handed and right-foot dominant healthy young subjects (1 female; age: 21–25; mean age: 22.6) took part in these experiments after providing written informed consent. Subjects had no history of neurological disorders and had not previously participated in a similar MI study. Our experimental paradigm was approved by ethics committee of Tokyo University of Agriculture and Technology and conducted in accordance with the Declaration of Helsinki.

### Experimental system

**Fig 1:**
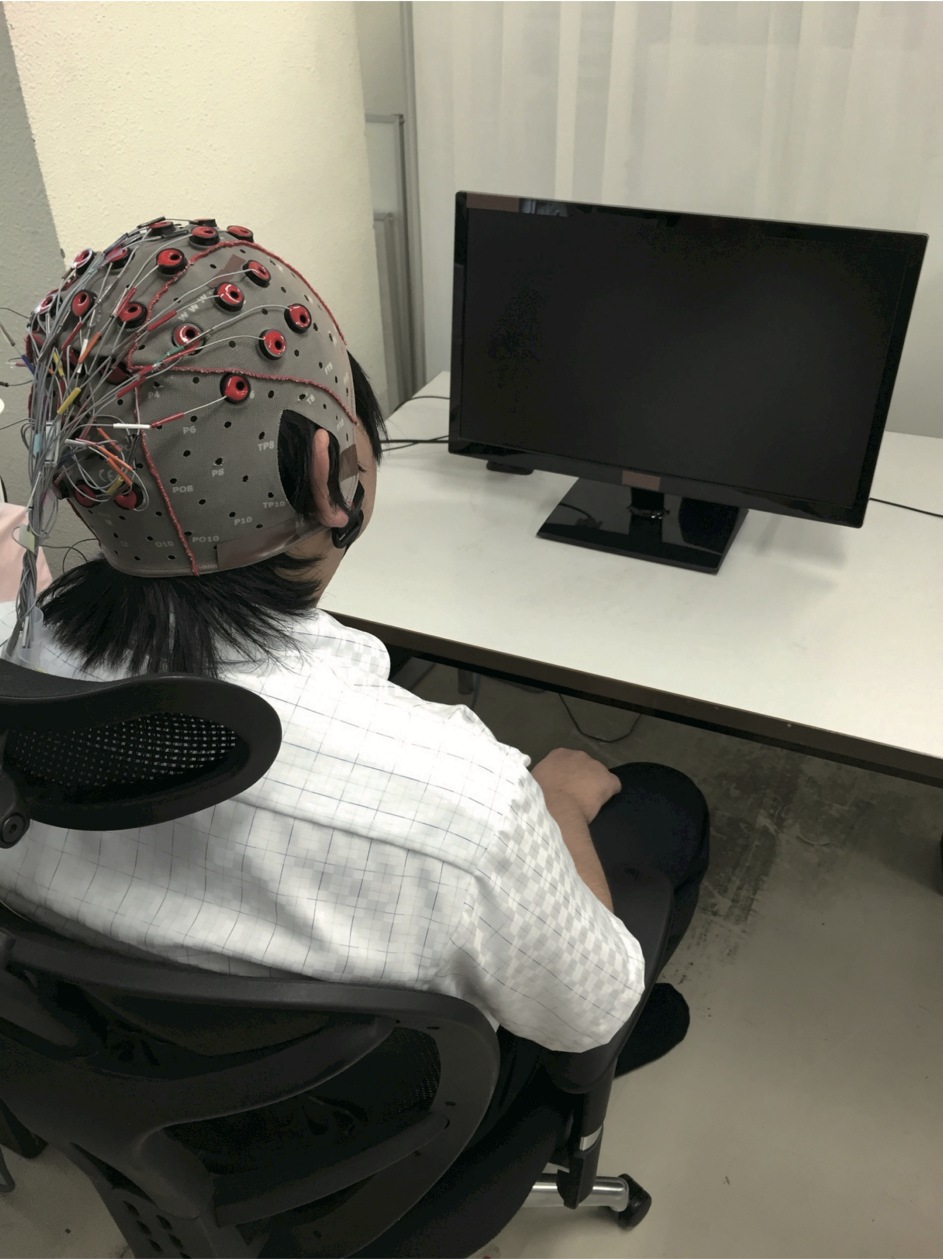
Experimental system. Subjects were seated in a high-back chair placed in front of a 24-inch LCD monitor displaying visual stimuli for specific experimental conditions. Stimuli included video clips of movements from the participant’s perspective and fixation cross.

**Fig 2:**
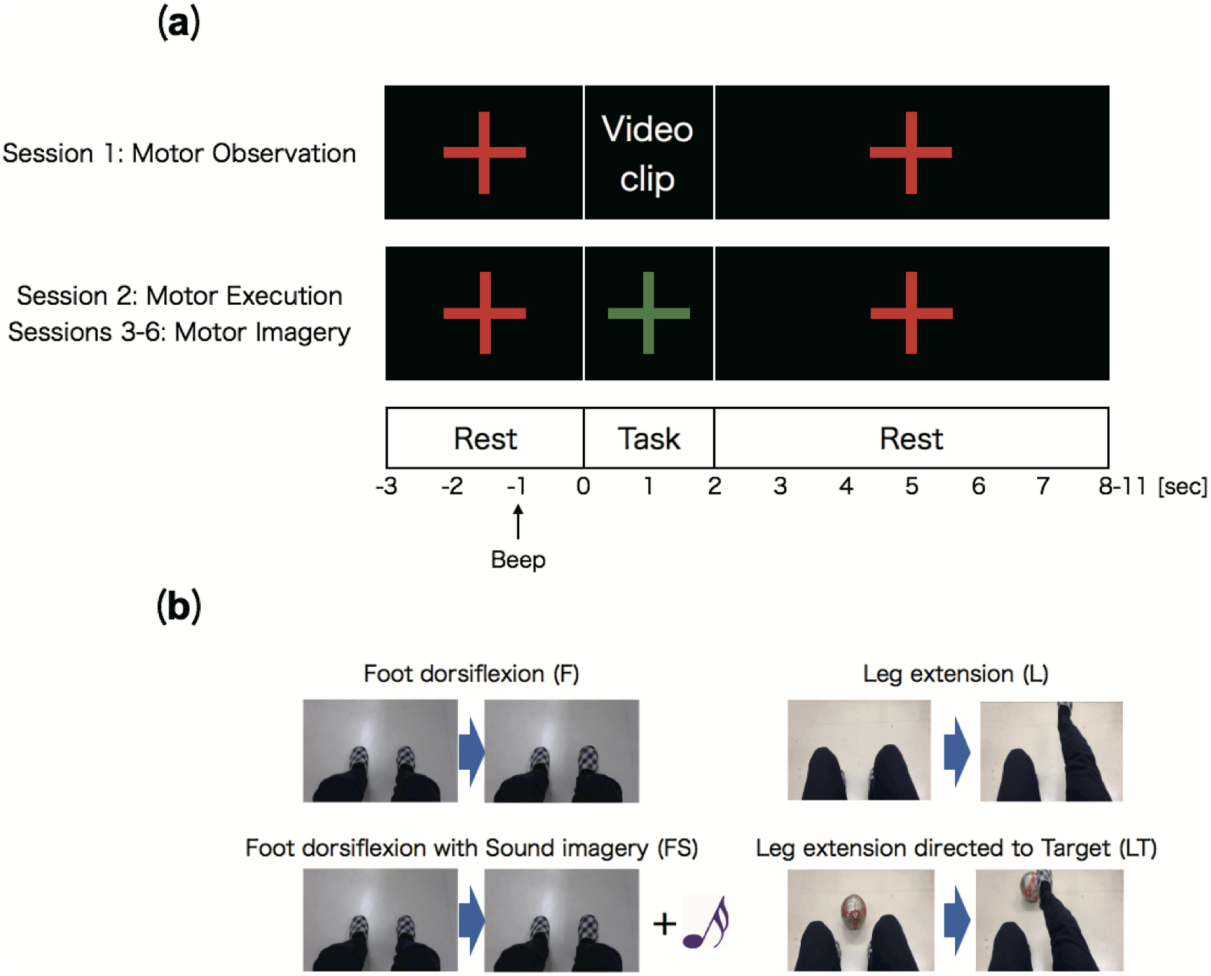
Experimental task. **(a) Flow of trial.** A trial was composed of a rest period (-3.0—0.0 sec) with a beep provided at -1.0 sec for task preparation, a task period (0.0—2.0 sec), and a rest period of random duration between 2.0—11.0 sec. There were 6 sessions for each experimental condition (**F**, **FS**, **L**, **LT**). In session 1, instructive video clips were displayed instead of a green fixation cross. In sessions 2–6, red and green fixation crosses were displayed on the monitor during the rest and task periods, respectively. **(b) Instructive video clips shown in session 1.** In condition **F**, subjects watched a video clip of foot dorsiflexion. In condition **FS**, subjects watched the same video clip of foot dorsiflexion but with the sound of a bass drum (a non-tonal sound) produced when the right sole touched the floor (the musical note is not displayed, visual information is the same as condition **F**. In condition **L**, subjects watched a video clip of leg extension. In condition **LT**, subjects watched a video clip of a ball being kicked using the same movement (i.e., target-directed motion).

The subjects were seated in a comfortable high-back chair in front of a 24-inch LCD monitor that displayed different visual stimuli (fixation cross and video clips) depending on the experimental condition (Figs 1 and 2).

**Fig 3:**
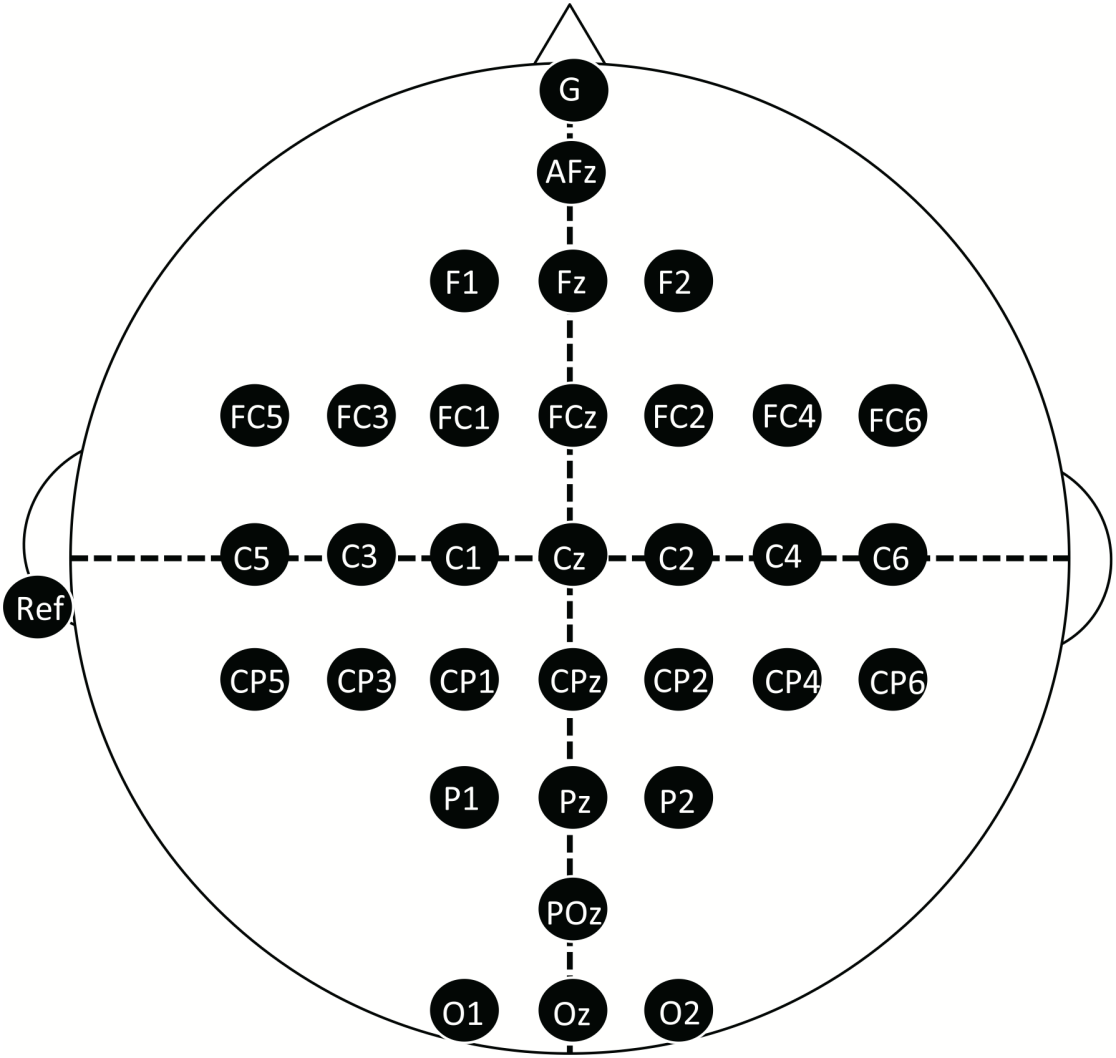
Electrode locations. EEG signals were recorded from 32 active EEG electrodes placed based on the international 10–20 system. “G” and “Ref” indicate the positions of ground and reference electrodes, respectively.

For EEG recording, subjects wore a cap with 32 active EEG electrodes (g.LADYbird, g.tec, Austria) covering the entire scalp with an electrode configuration based on the international 10–20 system (Fig 3). Ground and reference electrodes were placed on their forehead and left earlobe, respectively. EEG signals were amplified (sensitivity: 50 *μ*V/V) by using a AB-611J amplifier (NIHON KODEN, Japan) with band-pass filtering at 0.5—100 Hz, and digitized at a fixed sampling rate of 250 Hz (AD12–16, CONTEC, Japan). All digital EEG signals were stored on a personal computer (Windows 7 Professional, Intel Xeon CPU E5-2603 v3 1.60 GHz 2 processors). Further analyses of the recorded data were performed using MATLAB 2016a (The MathWorks, USA).

### Experimental design

To investigate the effects of target-directed movement and sound imagery on EEG sensorimotor rhythms during lower limb MI, we conducted experiments separated into the following two sections: **Sound imagery** and **Target-directed imagery**. Each section consisted of the following two conditions:

- **Sound imagery**
  - Condition **F**: subjects were asked to imagine right foot dorsiflexion as a control condition.
  - Condition **FS**: subjects were asked to imagine right foot dorsiflexion and the sound of a bass drum when the subject’s sole touched the floor.
- **Target-directed imagery**
  - Condition **L**: subjects were asked to imagine right leg extension as a control condition.
  - Condition **LT**: subjects were asked to imagine right leg extension directed to a soccer ball (i.e., target-directed movement).

All subjects experienced the four conditions separately with enough rest break among the conditions. Each condition consisted of the following six experimental sessions separated by a fixed break time (10.0 sec) with black screen.

- **Session 1 (Motor Observation)**: subjects were asked to watch an instructive video clip for each condition.
- **Session 2 (Motor Execution)**: subjects were asked to execute the actual lower limb movement without watching the video clip.
- **Sessions 3–6 (Motor Imagery)**: subjects were asked to perform kinesthetic MI of the instructed lower limb movement for each condition.

Each session included 10 repetitions of an experimental trial depicted in Fig 2a. Each trial consisted of a rest period (-3.0—0.0 sec, a warning beep provided at -1.0 sec), a task period (0.0—2.0 sec), and a rest period of random duration (2.0—11.0 sec). During the task period of the first session (i.e., Motor Observation session) for each condition, subjects were asked to watch an instructive video clip of the specific movement to be imagined in sessions 3—6 (Fig 2b) instead of the green fixation cross. During the task period in the second session (Motor Execution session), they were instructed to execute the actual lower limb movement without watching the video clip. In session 2 for conditions **F** and **FS**, subjects were asked to execute actual right foot dorsiflexion as practice for kinesthetic MI in the subsequent sessions, while in session 2 for conditions **L** and **LT**, they were asked to execute actual right leg extension. During the task period in sessions 3—6 (Motor Imagery sessions), the subjects were asked to perform MI of the instructed lower limb movement (i.e., foot dorsiflexion, foot dorsiflexion with the sound of a bass drum, leg extension, and kicking a ball) to evaluate ERD/S generation by MI. Throughout sessions 2—6, they watched a fixation cross without any sensory stimulus or feedback, while throughout session 1, visual and auditory stimuli (i.e., video clips) was presented in the task period. All subjects performed these four types of MI experiments, and EEG data obtained in sessions 3—6 were used for the analysis.

### Calculation for the ERD/S

We calculated ERD/S using the inter-trial variance (ITV) method (Eqs (1)–(3)) [27].

$$\begin{array}{c} A_{j}=\frac{1}{N-1}\sum_{i=1}^{N}y_{ij} \end{array}$$ (1)

$$\begin{array}{c} y_{ij}={\left(x_{ij}-\bar{x_{j}}\right)}^{2} \end{array}$$

$$\begin{array}{c} R=\frac{1}{|S_{R}|}\sum_{\forall j\in S_{R}}A_{j} \end{array}$$ (2)

$$\begin{array}{c} ERD_{j}=\frac{{\bar{A}}_{j}-R}{R}\times 100\left[\%\right] \end{array}$$ (3)

where *N* is the total number of trials, *x*_*ij*_ is the *j*th sample of the *i*th trial of the band-pass filtered EEG data, and $\bar{x_{j}}$ is the mean of the *j*th sample averaged over all trials. In this study, we defined the period from -3.0 to -1.0 sec before the task period as the reference period (baseline of RED/S). In Eq (2), *R* is the average power in the reference period, and *S*_*R*_ represents a set of samples within the period. Moreover, in this study, we calculated ERD/S only for the channel Cz (i.e. vertex area), because lower limb movements are represented in this cortical region [14]. The EEG data recorded in sessions 3—6 (i.e., 40 trials) were separated into 40 epochs by eliminating unnecessary rest periods and then subjected to common average derivation. As in [14], the derived EEG data were filtered by a 4th-order Butterworth filter with a band ±1 Hz from the center frequency. The center frequency was shifted from 3 to 45 Hz in intervals of 1 Hz. Using these 40 epochs, we calculated ERD/S in each frequency band based on the ITV method (i.e., *N* = 40). Note that after calculating *A*_*j*_ in ITV method, it was smoothed by simple moving average filter with a window of 250 ms (i.e., nearest preceding 62 samples) to estimate robust power change in each frequency band. It is denoted as ${\bar{A}}_{j}$. A bootstrap algorithm [28] was applied to identify significant ERD/S events relative to the baseline period. Only significant ERD/S events are shown in time-frequency maps (confidence interval; 99%).

## Results

### Time-frequency analysis of the ERD/S during MI

**Fig 4:**
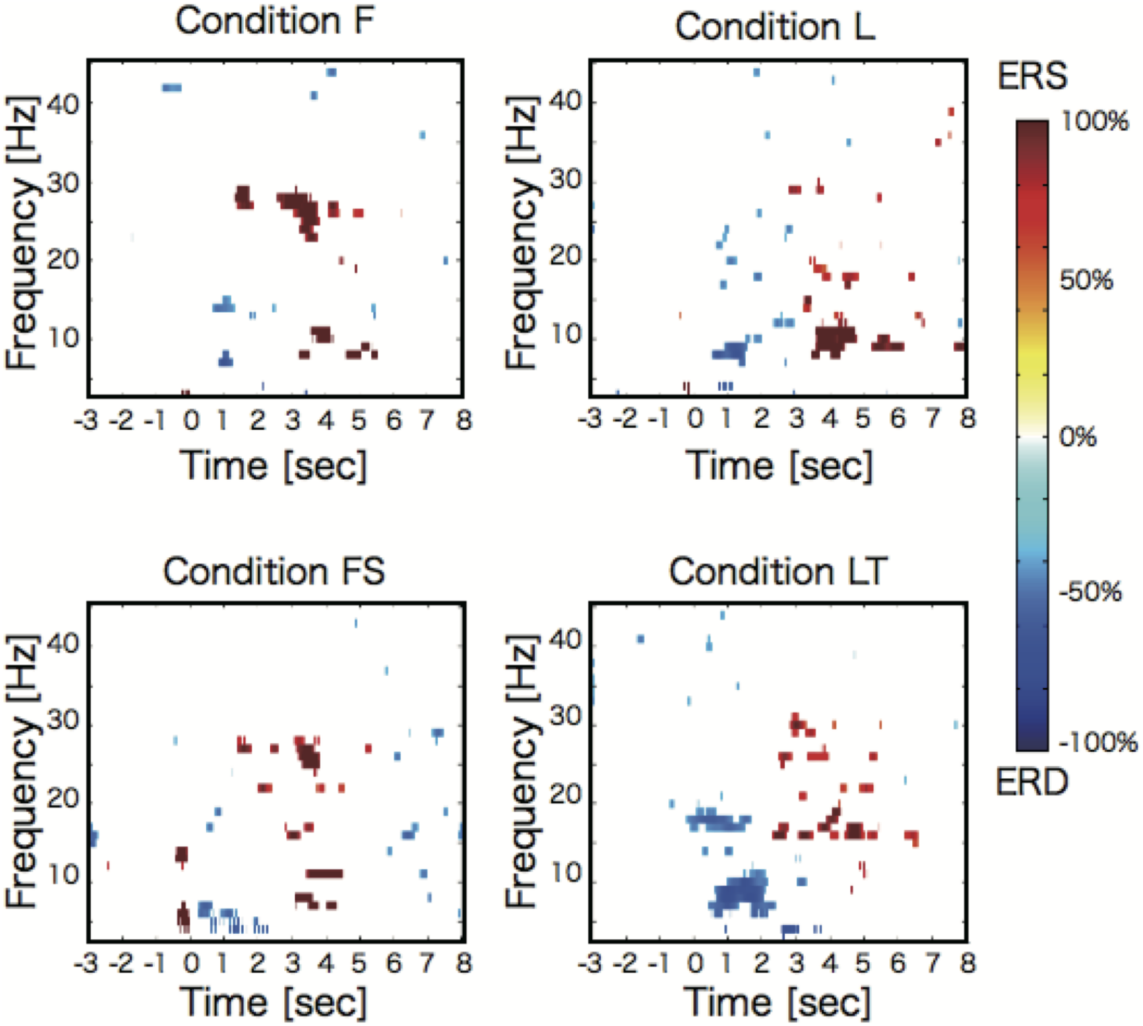
Time-frequency maps of ERD/S during sessions 3—6 (MI sessions). The upper left map shows the results for right foot dorsiflexion MI (condition **F**), the lower left map results for right foot dorsiflexion MI with sound imagery of a bass drum (condition **FS**), the upper right map results for right leg extension MI (condition **L**), and the lower right map results for target-direct right leg extension MI (kicking a ball, condition **LT**). Mental imagery to a target (**LT**) induced significantly larger ERD within the beta band than non-target MI (leg extension, **L**), while there were no significant differences in ERD/S between dorsiflexion MI with and without sound imagery (conditions **FS** and **F**).

Fig 4 shows the time-frequency maps of the ERD/S events during sessions 3—6 (MI sessions) for a representative participant (subject 9) under four conditions: right foot dorsiflexion MI (condition **F**), right foot dorsiflexion MI with sound imagery of a bass drum (condition **FS**), right leg extension MI (condition **L**), and right leg extension MI directed to a target (kicking a ball, condition **LT**). These time-frequency maps were calculated from the EEG signals recorded at Cz channels with a common average reference, and were derived only from significant ERD/S events (relative to baseline) as identified by a bootstrap algorithm [28]. As indicated in Fig 4, target-directed right leg extension MI was associated with stronger beta-band ERD than non-target leg extension MI (i.e., condition **LT** vs. **L**).

### Statistical comparison of the ERD/S

Peak values of mu-band ERD (8—13 Hz), beta-band ERD (14—30 Hz), and beta-band ERS (14—30 Hz) during sessions 3—6 (MI sessions) were obtained from the time-frequency maps for each subject. Previous studies established that ERD is induced during MI/ME/MO and ERS is induced after termination of MI/ME/MO. Thus, ERD and ERS were identified within the vicinity of the task period (0.0—3.5 sec) and the rest period after the task period (2.0—8.0 sec), respectively. These values are summarized in Table 1 (S1 File).

**Table 1:** The values of mu-ERD, beta-ERD, and beta-ERS during the sessions 3—6 (MI sessions).

	Mu-ERD											
	Condition F			Condition FS			Condition L			Condition LT		
ID	Value [%]	Frequency [Hz]	Latency [sec]	Value [%]	Frequency [Hz]	Latency [sec]	Value [%]	Frequency [Hz]	Latency [sec]	Value [%]	Frequency [Hz]	Latency [sec]
S1	-63.6	12	0.4	-60.2	10	0.2	-54.8	10	0.2	-55.3	9	1.6
S2	-46.8	9	0.6	-44.0	9	0.2	-50.5	10	0.8	-39.6	8	0.2
S3	-56.1	9	0.0	-62.9	10	0.1	-62.1	9	0.1	-39.1	13	0.1
S4	-43.0	12	1.0	-43.8	10	0.1	-41.3	10	0.0	-32.2	8	1.3
S5	-47.7	10	1.1	-46.3	13	1.9	-67.7	9	1.2	-56.7	8	1.1
S6	-50.2	10	3.1	-45.5	13	1.8	-57.3	10	1.4	-56.2	11	1.0
S7	-55.6	10	0.9	-64.8	11	0.9	-60.2	10	1.0	-66.1	9	1.1
S8	-40.1	9	0.4	-41.8	9	0.0	-43.3	11	0.9	-46.7	9	1.0
S9	-55.9	8	1.0	-50.6	8	0.5	-67.1	8	1.3	-77.0	9	1.0
Mean	-51.0	9.9	0.9	-51.1	10.3	0.6	-56.0	9.7	0.8	-52.1	9.3	0.9
S.E.	2.5	0.5	0.3	3.0	0.6	0.2	3.2	0.3	0.2	4.7	0.6	0.2
	Beta-ERD											
	Condition F			Condition FS			Condition L			Condition LT		
ID	Value [%]	Frequency [Hz]	Latency [sec]	Value [%]	Frequency [Hz]	Latency [sec]	Value [%]	Frequency [Hz]	Latency [sec]	Value [%]	Frequency [Hz]	Latency [sec]
S1	-52.0	27	0.8	-47.9	26	1.0	-40.0	20	2.4	-48.0	14	1.2
S2	-55.2	28	0.7	-49.2	16	2.9	-52.6	18	1.5	-59.7	23	3.5
S3	-53.5	29	0.3	-49.2	25	0.0	-41.0	14	2.7	-56.8	23	0.9
S4	-58.3	16	0.1	-56.9	27	0.3	-41.2	24	0.1	-58.8	16	1.0
S5	-50.3	26	2.8	-47.4	29	2.0	-40.1	14	2.5	-48.4	14	2.7
S6	-43.7	27	2.1	-43.7	27	2.2	-48.1	20	2.6	-43.2	14	3.3
S7	-41.6	19	3.0	-58.9	24	2.9	-44.1	24	2.9	-46.1	29	0.9
S8	-36.9	21	1.8	-48.3	22	2.0	-36.7	17	1.0	-47.0	27	0.1
S9	-52.0	14	0.9	-53.2	19	0.8	-49.5	18	1.9	-60.7	18	0.5
Mean	-49.3	23.0	1.4	-50.5	23.9	1.6	-43.7	18.8	2.0	-52.1	19.8	1.6
S.E.	2.3	1.9	0.4	1.6	1.4	0.4	1.8	1.2	0.3	2.3	2.0	0.4
	Beta-ERS											
	Condition F			Condition FS			Condition L			Condition LT		
ID	Value [%]	Frequency [Hz]	Latency [sec]	Value [%]	Frequency [Hz]	Latency [sec]	Value [%]	Frequency [Hz]	Latency [sec]	Value [%]	Frequency [Hz]	Latency [sec]
S1	242.0	21	3.4	214.1	23	3.3	151.2	23	2.9	87.6	14	3.3
S2	220.6	27	6.1	97.0	29	3.3	80.0	25	3.1	101.5	15	5.3
S3	146.2	25	3.9	168.7	27	3.6	65.1	28	3.4	72.2	30	2.9
S4	173.3	22	3.1	119.8	19	5.7	71.6	19	5.6	92.1	16	3.7
S5	104.0	27	6.4	72.1	23	8.0	96.9	28	7.7	49.8	17	3.6
S6	119.1	17	7.8	134.8	30	5.1	82.0	14	2.6	115.5	17	6.6
S7	77.6	15	3.2	56.8	14	4.8	73.6	14	4.8	101.0	14	3.5
S8	171.4	23	3.6	162.6	26	6.0	122.9	25	2.0	98.7	24	3.0
S9	146.9	27	3.0	199.9	26	3.5	110.3	15	3.3	135.4	16	2.5
Mean	155.7	22.7	4.5	136.2	24.1	4.8	94.8	21.2	3.9	94.9	18.1	3.8
S.E.	17.7	1.5	0.6	18.3	1.7	0.5	9.5	1.9	0.6	8.1	1.8	0.4

As shown in Table 1, all subjects except subject 6 generated larger beta-band ERD in condition **LT** than condition **L**, and all subjects generated larger beta-band ERS in condition **F** than condition **L**. There were significant differences in beta-band ERD/S among conditions. Specifically, MI directed to a target induced significantly larger ERD during the task period, while dorsiflexion elicited larger ERD than leg extension.

**Fig 5:**
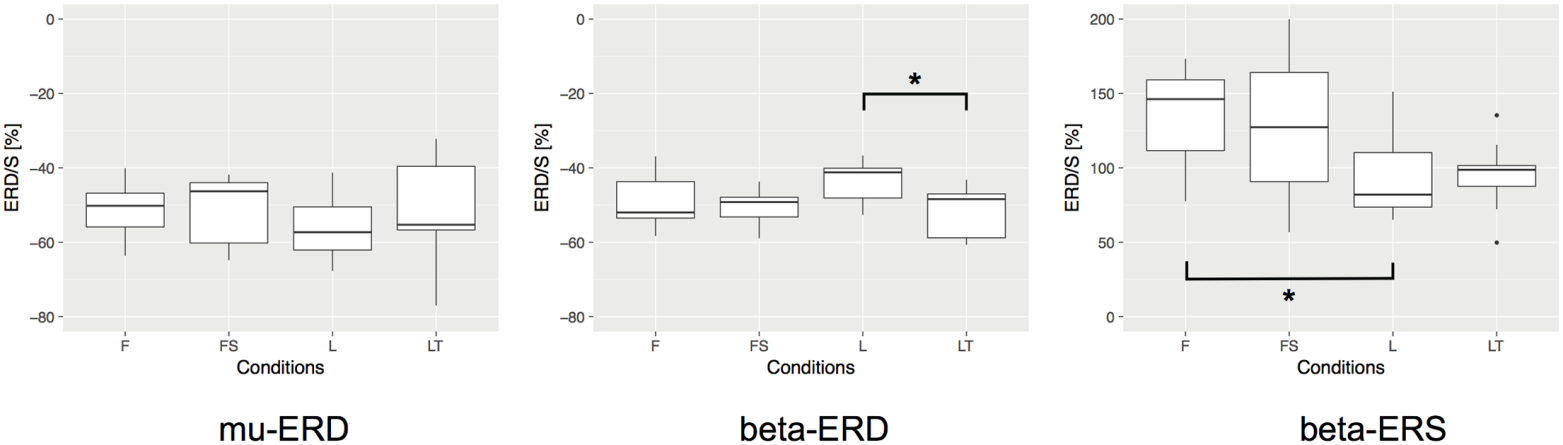
Statistical comparison of the ERD/S during the sessions 3—6 (MI sessions). We firstly performed one-way repeated measures ANOVA and secondly performed the post-hoc test by multiple comparison with Bonferroni correction to assess significant differences among MI conditions in mu-band ERD, beta-band ERD, and beta-band ERS. Asterisk between two conditions indicates a significant difference (*p* < 0.05, after correction).

To analyze the experimental data statistically, we firstly performed one-way repeated measures analysis of variance (ANOVA), and we found that the difference in MI condition significantly affected the level of the ERD generation in beta-band (*F*(3, 24) = 5.031, *p* < 0.01) and beta-band ERS (*F*(3, 24) = 7.305, *p* < 0.01). Secondly, post-hoc test by multiple comparison (Bonferroni correction) revealed that there was a statistical difference between conditions **L** and **LT** in beta-band ERD (*p* = 0.037, after correction), and conditions **F** and **L** in beta-band ERS (*p* = 0.024, after correction). Fig 5 shows the statistical comparisons mentioned above. In the figure, asterisk between two conditions indicates a significant difference (*p* < 0.05, after correction). To be clear the significance of our statistical tests against the sample size, in our case the number of the participants, we performed the post-hoc power analysis with the significance level of 0.05, and found that the power of our test was 0.92 for beta-band ERD and 0.98 for beta-band ERS. Thus, we can conclude that our results were statistically validated. These statistical comparisons revealed significant differences between conditions **L** and **LT** in beta-band ERD and conditions **F** and **L** in beta-band ERS, but no significant differences were confirmed among MI conditions in mu-band ERD. In addition, there were no significant differences in the latency and frequency distribution between the MI conditions for mu-band ERD, beta-band ERD, and beta-band ERS.

These results suggest that having a target during lower limb MI strengthens beta-band peak ERD compared to the non-target condition. Furthermore, the foot dorsiflexion MI generates a significantly larger beta-band ERS than leg extension MI.

## Discussion

In the current study, we demonstrated that target-directed MI could produce the larger ERD in beta-band compared to non-target MI (*p* = 0.037, after correction). A previous study [21] reported that watching goal-oriented visual stimuli during MI could enhance ERD, but their study did not evaluate whether the target-directed MI alone can enhance ERD/S compared to the non-target MI condition. Thus, we expect that our result of the pure target-directed MI should have more direct relevance to the online BCI control, as it does not require the visual stimuli, addressing an active BCI control rather than passive BCI. In the latter, a certain brain state is simply induced by the particular visual stimulation.

As one of the possible explanations of our main result, the target-directed MI would be accompanied by the ‘imagined’ tactile feedback signals in the brain, as the subject imagined a consequence of their lower limb motion, effectively ‘kicking a ball’. This creates an opportunity for the MI to include the imagery of motion as well as the sensory feedback by closing the loop between brain and body, which might resulted in strengthening ERD. This interpretation is supported by two previous discoveries; 1. the tactile stimuli should enhance ERD/S [29]. 2. the magnitude of grasping force of a hand did not make a difference in the strength of ERD/S [30]. The second point is derived from a conclusion on actual execution of motion, but we speculate that imagining ‘stronger’ motion would not help the stronger ERD.

More generally speaking, what is the benefit of using the goal-oriented tasks in BCI? A BCI output pathway can function in two different ways: it can control a process or it can select a goal [31, 32]. In the former, subjects are asked to imagine a certain motion, which would specify each of the sequence of motion to control the output device. In the latter, the output pathways provided by a BCI system can simply communicate with the goal (e.g. the target to which the cursor should move). Wolpaw reported that many non-invasive and most of invasive BCI studies have adapted the process-control strategy, while just a few non-invasive BCI studies, for example, using the P300 evoked potential have adapted the goal-selection strategy. The general argument is such that the process-control places greater demands on the BCI performance than the goal-selection, as the process-control requires that the cortex provide the rapid responses to position-, velocity-, and acceleration-related planning, thus the as a means to establish the communication channel, it would be harder for any subjects to sustainably imagine the same motion in terms of kinematic information flows. Alternatively, the goal-selection process would be easier. Upon the BCI operation, it only requires the user’s intent to choose the goal without requiring the lower level motor planning. Following this comprehensive discussion of process-control and goal-control, we considered that the experimental paradigm of target-directed motor imagery should be related to the goal-selection process. Much of attention will be generated around the visual target on the display, when the subject is engaged in the motor imagination tasks. Thus, we expect the higher performance in the target-directed motor imagery BCI.

The present study also found that foot dorsiflexion induced significantly larger beta-band ERS than leg extension (*p* = 0.024, after correction). A previous study focusing on upper limb movement found that BCI performance was for proximal movement could be better than for distal movement [33], while another study found that shoulder movements (proximal joint movement) activated a wider area of cortex than hand movements (distal joint movement) [34]. In contrast, our results indicate that distal joint movement (foot dorsiflexion) generates larger beta-band ERS than proximal joint movements (leg extension). Therefore, in addition to differences between upper and lower limbs, there appear to be difference in ERS properties when evoked by different joints within the same limb.

## Conclusions

We conducted the experiments to address three questions regarding motor imagery of the lower limb: 1) whether target-directed MI affects (particularly enhances) ERD/S, 2) if MI with sound imagery affects ERD/S, and 3) whether ERD/S differs between ankle and knee movements. We confirmed that beta-band ERD was significantly strengthened by a target for MI compared to no target. This is consistent with a previous study reporting that observing upper arm movement directed to a target during MI strengthened ERD generation [20]. Note that our previous study suggested that force of movement does not affect the strength of ERD [30]. Therefore, we conclude that this ERD enhancement is caused by the (imaginary) target. Furthermore, a previous study [21] indicated that lower limb MI while watching goal-oriented visual stimuli produced stronger ERD, while we found that target-directed MI without actual visual stimuli also enhanced beta-band ERD compared to non-target MI. This feature could be crucial for the development of practical BCIs.

In regard to the third question, beta-band ERS induced by foot dorsiflexion (distal joint movement) was significantly larger than those generated by leg extension (proximal joint movement). In contrast, a previous study found that BCI performance was superior for proximal joint movement compared to distal joint movement [33], and that proximal joint movements should modulate a greater areas of cortex than distal joint movements [34].

We conclude that target-directed MI should cause larger beta-band ERD than non-target MI. In a previous study [35], target-directed MI improved BCI accuracy compared to non-target MI in healthy participants with low kinesthetic MI scores according to the KVIQ [36], while BCI accuracies deteriorated in the target-directed condition for participants with high kinesthetic MI scores. In the present study, all subjects were relatively naive, so target-directed MI should indeed have induced larger beta-band ERD than non-target MI. We suggest that the appropriate MI condition is critical for optimal MI control of BCIs, both for onsite neurorehabilitation and for use in daily life.

## Supporting information

S1 File The peak values of mu-ERD, beta-ERD, and beta-ERS during the sessions 3—6 under each experimental condition. These are identical to the data listed in Table 1.
(XLSX)
